# The gut–bone axis in osteoporosis: microbial mechanisms, conflicting evidence, and translational priorities

**DOI:** 10.3389/fimmu.2026.1966537

**Published:** 2026-10-14

**Authors:** Zhe Liu, Chunli Yu

**Affiliations:** 1 The Second Clinical School of Ningxia Medical University‌, Yinchuan, Ningxia, China; 2 Orthopedics Department of The First People’s Hospital of Yinchuan, Yinchuan, Ningxia, China; 3 Intensive Care Unit of Shaanxi Provincial Nuclear Industry 215 Hospital, Xianyang, Shaanxi, China

**Keywords:** bone mineral density, gut microbiota, gut–bone axis, osteoimmunology, osteoporosis, probiotics, short-chain fatty acids, translational microbiome research

## Abstract

Microbial effects on bone depend on the host’s hormonal and inflammatory state, the microbial exposure and the tissue that receives it. This critical narrative review examines the gut–bone axis through those relationships, separating microbial associations from tested mechanisms and product-specific clinical effects. In mice, short-chain fatty acids can suppress osteoclast differentiation through metabolic reprogramming or promote bone formation through regulatory T-cell control of WNT10B production by CD8+ T cells. Intestinal T-cell trafficking also contributes to trabecular bone loss in an estrogen-deficiency model. These experiments identify causal links within defined systems; they do not establish equivalent pathways in patients. Bile acids, indole derivatives and bacterial extracellular vesicles provide further candidates, although evidence for tissue exposure and immune mediation varies. Human cohorts associate microbial features with bone density and incident fracture, but no consistent taxonomic signature has emerged across populations. Functional profiles may aid mechanistic interpretation, but their reproducibility across populations remains uncertain. Probiotic and synbiotic trials report benefit, no detectable effect or greater bone loss at particular sites. Discordance persists even between studies using the same strain accessions, making population, formulation and endpoint essential to interpretation. We distinguish microbial target engagement from bone-turnover responses, structural skeletal measures and fracture outcomes. The clinical evidence reviewed is insufficient to establish that microbiota-directed interventions reduce fragility-fracture risk. The recent, context-specific qualification of total-hip bone density as a surrogate nevertheless matters for future trial design. The most useful next studies will measure exposure and host response together, test the necessity of proposed mediators and prospectively examine host factors that may modify treatment response. This approach identifies where microbial mechanisms could complement established osteoporosis care without treating biological plausibility as clinical efficacy.

## 1 Introduction

Osteoporosis weakens bone through loss of mass and deterioration of tissue architecture. Its clinical burden is concentrated in fragility fractures and their consequences for mobility, independence and survival (1, 2). Postmenopausal, age-related, glucocorticoid-associated and inflammatory bone loss arise in different biological settings. This variation is relevant to the gut microbiota because host physiology shapes both intestinal ecology and the skeletal response to microbial products.

Bone remodeling couples osteoclast-mediated resorption to osteoblast-mediated formation within a network of osteocytes, stromal cells and immune cells. Osteocytes and osteoblast-lineage cells help regulate osteoclast development through receptor activator of nuclear factor-κB (RANK), its ligand (RANKL) and the decoy receptor osteoprotegerin (OPG). Immune-cell cytokines alter this local environment, but a circulating concentration does not describe the whole remodeling unit (3, 4). Estrogen withdrawal, ageing, glucocorticoids and nutritional deficiency can disturb this coupling through different combinations of cellular and systemic effects.

Current care combines calcium and vitamin D sufficiency with antiresorptive or anabolic treatment chosen according to fracture risk. These medicines reduce fractures, although adherence, treatment sequence, rebound after denosumab and uncommon adverse events require active management (1, 2). Microbiota-directed approaches should therefore be judged as possible adjuncts to effective therapy, not as replacements for it.

The intestinal microbiota contributes to nutrient transformation, colonization resistance and immune development. Its effects depend on community interactions as well as the identity of individual organisms. Diet, medication, age and host physiology influence both microbial composition and the products available to host tissues (5–7). A taxonomic difference is therefore a starting observation, not a specified immune exposure. For mechanistic interpretation, it must be connected to a microbial molecule, antigenic stimulus or ecological function.

Gut inflammation offers a useful example of this complexity: systemic inflammation, malnutrition and treatment can each contribute to bone loss, alongside possible microbial effects (5–7). Recent reviews have mapped microbial metabolites, brain–gut signaling and other gut–organ connections in bone disease (8–10). A more discriminating question is whether a proposed microbial signal, its host target and the skeletal response have been demonstrated in the same experimental or clinical setting.

We examine which microbial exposures alter immune or skeletal targets, where the evidence extends to humans and what explains discordant results. Receptor engagement and tissue localization are central to this analysis. Comparing a direct osteoclast effect with intestinal immune-cell trafficking, for example, requires different evidence even when both change bone mass. The review uses such distinctions to identify missing causal links and propose tests that could resolve them.

The evidence is organized by mechanism and then by human study design and intervention. Positive findings are considered alongside null results and model-specific counterexamples. **Table 1** summarizes the questions each design can answer; the subsequent appraisal separates ecological interventions from defined strains, metabolites and delivery systems.

**Table 1:** How different study designs inform the gut–bone axis.

Study design	Question it can address	Key credibility conditions	Inference it cannot supply alone
Cell/animal perturbation	Does a specified exposure or mediator change a bone phenotype within this model?	Tissue exposure; matched controls; necessity/sufficiency tests; model and experimental-unit checks	Human efficacy, achievable human dose or fracture prevention
Cross-sectional human study	Which microbial, immune and skeletal measures coexist?	Assay resolution; compositional analysis; justified confounders; external replication	Temporal order, tissue trafficking or mediation
Prospective cohort	Does an exposure precede the skeletal outcome?	Repeated measurements; outcome ascertainment; time-varying confounders and competing events	Exclusion of residual confounding or a specific immune mechanism
Mendelian randomisation	Are genetically proxied exposure–outcome relations compatible with a causal hypothesis?	Relevant instruments; independence; exclusion restriction; sensitivity and ancestry checks	Reliable direction if assumptions fail; equivalence to strain supplementation
Randomised product trial	Does this product change the prespecified endpoint in this population?	Allocation; identity/dose; adherence; attrition; precision; safety; sufficient follow-up	Class-wide efficacy or mediation without an appropriate mechanistic design
Evidence synthesis	How consistent are comparable study estimates?	Underlying study quality; endpoint compatibility; heterogeneity; trial-overlap assessment	Independent replication when the same trials recur; correction of primary-study bias

## 2 Review design and evidence appraisal

### 2.1 Review design and scope

This article is a critical narrative review of microbial mechanisms, human associations and microbiota-directed interventions relevant to osteoporosis. The synthesis draws on primary experiments, observational studies, prospective cohorts, Mendelian randomization, randomized trials and published evidence syntheses. These designs answer different questions. We distinguish evidence for association, temporality, experimentally tested mediation and intervention efficacy without combining them into a single causal hierarchy. No new participant-level analysis or *de novo* meta-analysis was performed.

### 2.2 Literature search strategy

The initial literature search was conducted on 1 December 2025 and updated on 20 July 2026. The databases were PubMed, including MEDLINE, Web of Science Core Collection, Scopus and the Cochrane Library. Coverage extended from the available database records to the July update. Zhe Liu performed the searches, and Chunli Yu reviewed the search strategy. English-language full texts were required for detailed appraisal.

The search record grouped terms into intestinal microbiota, skeletal outcomes, and mechanisms or interventions. **Supplementary Material S1** records the original settings and presents technical revisions: a two-domain core and separate product, mediator and upstream-mechanism searches. The revised architecture avoids requiring every relevant study to contain a mechanism term; it is distinguished from the earlier recorded formulations. Citation tracing and targeted journal searches supplemented database identification. S1 also distinguishes direct skeletal evidence from upstream mechanistic and contextual sources.

Reference-led checks on 19–20 September 2026 addressed reviewer-recommended articles, disputed mechanisms and named clinical products. Bibliographic and selected full-text information was checked using Crossref, PubMed/Europe PMC and publisher or official sources.

### 2.3 Eligibility and evidence synthesis

Direct primary evidence linked a microbial exposure or intervention to a quantitative skeletal outcome. Outcomes included areal or volumetric bone mineral density (BMD), bone mineral content, microarchitecture, strength, turnover markers and incident fracture. Mechanistic experiments were considered when they tested a microbial exposure, receptor, cell population or mediator in a bone-relevant model. Upstream studies of mineral absorption, epithelial function, immune regulation or endocrine signaling could support a proposed route without demonstrating a skeletal benefit. Such studies were treated as mechanistic context, not evidence of osteoporosis treatment efficacy. Background reviews, reporting guidance and regulatory sources served distinct explanatory purposes. Meta-analyses were interpreted alongside their constituent trials rather than as independent replications. Sample size informed appraisal of precision and bias, not a rigid exclusion threshold.

Two researchers independently screened titles and abstracts, followed by full-text assessment against the eligibility criteria. Disagreements were resolved through discussion, with third-person adjudication available if required. Selection for this critical narrative synthesis prioritized mechanistic relevance and findings that explained disagreement, including positive, null and unfavorable results. The extracted information covered study design, population or model, microbial exposure, tissue compartment, measured mediator, skeletal endpoint, main finding and interpretive limitation. **Table 2** separates primary human studies from evidence syntheses, while **Supplementary Material S2** maps selected mechanisms to their experimental support. The review did not produce a *de novo* meta-analysis or a numerical PRISMA flow diagram.

**Table 2:** Selected primary human studies and evidence syntheses: measured outcomes and limits of inference.

Study/population	Exposure, assay and endpoint	Principal finding	Interpretive boundary
A. Primary human observational studies			
Das et al. (34); 181 older adults	Faecal 16S profiles; DXA BMD	No alpha-diversity association with BMD; selected genus associations	Relative composition; diet, medication and health covariates matter
Ling et al. (38); 1,776 adults	Microbiome and faecal/serum metabolomics; osteoporosis status	Amino-acid-related associations	Cross-sectional mediation is not source attribution or temporal proof
Okoro et al. (33); FHS 1,227 and MrOS 836	16S profiles; density, microarchitecture and strength estimates	Few genus associations replicated; predicted functions discordant	Predicted function is not expression or metabolite flux
Gao et al. (35); 605 adults	Shotgun metagenomics; BMD and polygenic risk	No global diversity difference; species signals varied by genetic context	Relative species/gene profiles, not proof of causal microbial expansion
Li et al. (31); 684 adults plus validation	Shotgun metagenomics; DXA BMD	Selected taxa and functional pathways associated with BMD	Neither enterotype nor gene abundance proves mediation
Ma et al. (15); 77 postmenopausal women	16S profiles; peripheral Th17/Treg; serum cytokines/LPS	Immune and microbial measures differed by bone status	No gut-to-marrow cell tracking; LPS is not a direct permeability test
Grahnemo et al. (44); FINRISK, 7,043 adults	Shotgun profiles; 1,092 incident fractures; median 18 years	Prospective associations after covariate adjustment	Fractures were not an intervention outcome; residual confounding remains
B. Randomized trials: site-specific imaging outcomes, not observed fracture reduction			
Nilsson et al. (46); 90 women aged 75–80; 70 completed; 12 months	*Limosilactobacillus reuteri* ATCC PTA 6475, 10^10^ CFU/day; primary tibial total vBMD	ITT between-group difference in relative change: +1.02 percentage points (95% CI 0.02 to 2.03)	Small trial in older women with low BMD; secondary ITT outcomes generally non-significant
Jansson et al. (47); 249 randomised, 234 in primary analysis; 12 months	*L. paracasei* DSM13434 and *L. plantarum* DSM15312/DSM15313, 10^10^ CFU/day; lumbar-spine BMD	Difference in relative change: +0.71 percentage points (95% CI 0.06 to 1.35)	Primary analysis required baseline and 12-month scans; spine-specific finding; funded by Probi
Gregori et al. (48); 239 early postmenopausal women; 24 months	*Limosilactobacillus reuteri* ATCC PTA 6475; 10^9^ or 10^10^ CFU/day; primary tibial total vBMD; vitamin D in all arms	High dose vs placebo: −0.08 percentage points (95% CI −0.85 to 0.69); low dose: −0.22 (−0.99 to 0.55)	No statistically detectable benefit on prespecified endpoints; BMI interaction requires independent testing
Resciniti et al. (49); 114 early postmenopausal women; 12 months	*L. plantarum* DSM15312/DSM15313 and *L. paracasei* DSM13434, 10^10^ CFU/day; primary distal-tibial total vBMD	Between-group difference: −3.7 mg HA/cm³ (95% CI −6.9 to −0.4), favoring placebo	Greater loss at the primary site, not demonstrated fracture harm; most secondary outcomes non-significant after correction
Schott et al. (50); 286 non-osteoporotic early postmenopausal women; 221 completed; 12 months	SBD111, 9.5 × 10^10^ CFU/day plus substrates; primary lumbar-spine areal BMD	Between-group effect: +0.15 percentage points (95% CI −0.52 to 0.82)	Null primary result; subgroup estimates do not establish overall efficacy or microbial mediation
Liu et al. (51); 44 enrolled, 31 retained; 3 months	*L. fermentum* CCFM1126, 5 × 10^9^ CFU twice daily; DXA-derived T- and Z-scores	Favourable score changes; no site-specific absolute BMD effect with CI supplied in the extracted results	Small retained sample; unclear allocation/withdrawal chronology; three months; skeletal site insufficiently specified
C. Published syntheses: not independent replications of their constituent trials			
Wang et al. (52); 12 RCTs, 1,183 women	Probiotics; spine/hip BMD and turnover	Pooled favorable site-specific estimates and lower CTX-I	Product and population heterogeneity; overlapping trial base
Hidayat et al. (53); adult RCT synthesis	Probiotics; BMD, P1NP and CTX-I	Lumbar signal; hip estimate sensitive to exclusions	Heterogeneity; not evidence of fracture prevention
Gong et al. (54); 10 trials	Probiotic/synbiotic supplementation; lumbar-spine BMD	SMD 0.86 (95% CI 0.01 to 1.71); no significant total-hip effect	SMD is not percentage BMD gain; imprecise site-specific effect
Hamedi-Kalajahi et al. (19); animal interventions and human RCTs	RANKL-related outcomes	Clearer modulation in animal models than in human studies	Mixed-species synthesis; mechanistic readout is not a clinical endpoint

BMD, bone mineral density; BMI, body mass index; CTX-I, C-terminal telopeptide of type I collagen; vBMD, volumetric BMD; DXA, dual-energy X-ray absorptiometry; ITT, intention-to-treat; CFU, colony-forming units; CI, confidence interval; SMD, standardized mean difference; HA, hydroxyapatite; FHS, Framingham Heart Study; MrOS, osteoporotic fractures in men study; LPS, lipopolysaccharide; P1NP, procollagen type I N-terminal propeptide; RANKL, receptor activator of nuclear factor-κB ligand; RCT, randomized controlled trial; Th17, T helper 17; Treg, regulatory T cell. BMD changes in percentage points, mg HA/cm³ and standardized T- or Z-scores are not interchangeable. Estimates are taken from the specified primary endpoints, not recomputed or pooled. A non-significant result does not establish equivalence. Meta-analyses may share trials and should not be counted as independent replication. Product, dose and source-location notes are provided in **Supplementary Material S2**.

### 2.4 Critical appraisal

Appraisal used design-specific questions rather than a numerical quality score. For trials, these concerned allocations, verified product identity, prespecified outcomes, attrition, precision, adherence, follow-up and safety. For observational studies, they concerned temporal ordering, sampling, confounder control, compositional analysis and external replication. Mendelian randomization was interpreted only in relation to instrument relevance, independence and exclusion restriction, with attention to pleiotropy, ancestry and host-mediated pathways. Animal experiments were considered for model fit, dose, tissue exposure, experimental unit, receptor or mediator perturbation and independent replication. Mechanistic strength within an animal model was kept separate from direct applicability to patients. The Strengthening the Organization and Reporting of Microbiome Studies (STORMS) checklist informed the assessment of microbiome reporting, not a claim that all included studies met every item (11).

### 2.5 Methodological limitations of this review

Narrative selection and restriction to English-language full texts may introduce selection and language bias. Database indexing, differences in terminology and selective publication can also affect which studies are identified. Formal risk-of-bias instruments were not applied to every article; the design-specific appraisal therefore does not provide an all-study certainty grade. Detailed search specifications improve methodological clarity but do not establish exhaustive coverage. We retain conflicting findings and restrict causal and clinical conclusions to the designs, exposures and outcomes that support them.

## 3 The gut–bone axis: an overview of mechanistic pathways

The gut–bone axis describes communication between the intestinal ecosystem and skeletal tissues, not a single pathway shared by every form of osteoporosis. Germ-free, colonization and sex-steroid-deficiency experiments show that microbial exposure can alter bone mass and remodeling in defined settings (12–14). A mechanistic account must identify the microbial exposure, its product or function, a host receptor or immune cell, the relevant tissue compartment and the skeletal phenotype. Evidence for one link cannot substitute for the others. **Supplementary Material S2** applies this framework to selected pathways and records where causal attribution remains incomplete.

### 3.1 Immune modulation: the microbiota–immune–bone axis

Intestinal microbes continuously shape mucosal immunity, although many of these signals are unlikely to affect the skeleton. A plausible pathway must therefore be spatially as well as cellularly coherent, connecting a defined microbial exposure with an immune mediator, its movement or systemic consequence, and a change in the bone remodeling compartment.

#### 3.1.1 T-cell-mediated regulation: Th17/Treg balance

T helper 17 (Th17) cells and regulatory T (Treg) cells provide an immune link between microbial exposure and bone, but their ratio is not a universal functional index. Cytokine production, activation state, antigen specificity and tissue localization matter alongside cell frequency. Foxp3-positive Treg populations can restrain inflammatory responses, whereas Th17-associated interleukin-17 (IL-17) and tumor necrosis factor-α (TNF-α) can support osteoclastogenic conditions. These populations are not fixed, uniformly antagonistic compartments. A peripheral-blood shift cannot establish either an intestinal origin or an equivalent change in marrow (3, 13, 15).

Evidence for tissue-to-tissue communication comes from an ovariectomy model in which blocking lymphocyte egress or marrow recruitment limited marrow T-cell expansion and trabecular bone loss (16). The experiments implicated sphingosine-1-phosphate receptor 1 (S1PR1) in egress, C–X–C motif chemokine receptor 3 (CXCR3) in recruitment of TNF-positive T cells, and C–C motif chemokine ligand 20/receptor 6 (CCL20/CCR6) in Th17 recruitment. Protection did not extend equally to cortical bone. This compartment-specific result supports a trafficking mechanism in the model while leaving its contribution to human osteoporosis unresolved.

Estrogen withdrawal also changes the barrier and inflammatory context. In colonized mice, sex-steroid deficiency increased permeability and osteoclastogenic signaling, whereas germ-free status or specified probiotic treatment altered the trabecular response (13). Germ-free animals nevertheless differ in immune development and physiology, so the comparison alone does not isolate one microbial mediator. Direct tracking, trafficking blockade and soluble-mediator perturbation answer different questions and should not be treated as interchangeable evidence.

Human support is narrower. A study of 77 postmenopausal women combined fecal profiling with peripheral-blood Th17/Treg measurements and circulating inflammatory markers (15). It did not track immune cells from intestine to marrow. Elevated lipopolysaccharide (LPS) was an indirect exposure measure, not direct proof of epithelial permeability or bacterial translocation. Separately, control-donor fecal transfer improved bone microarchitecture, junctional-protein measures and microbial metabolic profiles in ovariectomized mice (17). That study did not measure Th17/Treg populations and cannot substantiate their normalization in gut or marrow.

#### 3.1.2 Innate immune states and microbial ligands

Innate responses depend on the ligand and receiving cell. Lipopolysaccharide, peptidoglycan-derived products, microbial nucleic acids and vesicle cargo engage different recognition systems; ‘dysbiosis’ does not specify which exposure changed. Receptor expression alone is insufficient to establish receptor dependence. Likewise, macrophage states extend beyond a binary M1/M2 classification, and shifts in selected markers need not mediate the skeletal response. The indole experiments discussed below are instructive because intestinal aryl hydrocarbon receptor (AhR) perturbation provides stronger localization evidence than macrophage-marker changes alone (18).

#### 3.1.3 The RANKL/RANK/OPG axis

RANKL binding to RANK is central to osteoclast differentiation, while OPG limits ligand availability as a soluble decoy receptor (3, 4). Relevant RANKL sources include osteocytes, osteoblast-lineage cells and immune populations, whose contributions depend on the disease setting. Local presentation, receptor availability and OPG buffering determine the signal received by a precursor. Circulating RANKL, tissue expression and a RANKL/OPG ratio are therefore related but non-equivalent measures. Their interpretation is stronger when accompanied by osteoclast differentiation or resorption assays and a skeletal endpoint. A review of probiotic interventions found clearer RANKL effects in animal models than in human trials (19). Neither altered expression nor a ratio establishes fracture protection.

### 3.2 Microbial metabolites: signaling molecules from gut to bone

Microbial metabolites may act at the mucosa, through circulating immune or endocrine signals, or directly on skeletal cells. A direct route requires evidence that the active exposure reaches the relevant cells. An indirect route requires identification of the intervening host response, but not necessarily transport of the microbial molecule to bone. Reviews provide context for these possibilities (6, 10); experimental attribution depends on what each study actually measured or perturbed.

#### 3.2.1 Short-chain fatty acids

Short-chain fatty acids (SCFAs), including acetate, propionate and butyrate, arise from microbial fermentation. Functional genes indicate production potential, whereas fecal concentrations reflect production together with consumption, absorption and transit. Neither establishes the dose received by skeletal cells. Luminal, portal, peripheral and marrow concentrations describe different exposures (6, 20). These distinctions apply equally to bile acids and indole derivatives.

Lucas et al. showed that propionate and butyrate altered osteoclast-precursor metabolism and suppressed differentiation (20). The response persisted without G protein-coupled receptors 41 and 43 (GPR41/43), and inhibition of the early glycolytic response counteracted suppression. The experiment therefore supports a metabolic route that cannot be reduced to obligatory GPR43 signaling. Histone deacetylase (HDAC) inhibition is another action of SCFAs, but invoking it does not identify the mechanism unless its contribution is tested.

Tyagi et al. identified a separate anabolic route: butyrate increased Treg populations, which regulated Wnt family member 10B (WNT10B) production by marrow CD8+ T cells (21). Treg depletion and adoptive-transfer experiments involving CD8+ cells lacking WNT10B disrupted the response. Tregs regulated this pathway; they were not the assigned WNT10B source. Related mouse experiments implicated microbial butyrate in the response to intermittent parathyroid hormone (PTH) (22). Whether this interaction changes treatment response in patients remains to be tested.

Ligand and cell context matter. In another study, *Gpr41/43* double-knockout mice had increased trabecular bone, while acetate favored adipogenic over osteogenic differentiation in wild-type marrow-derived cells (23). This is a counterexample to uniformly protective SCFA signaling, not a refutation of the propionate and butyrate experiments. The next translational question is which exposure and responding cell account for the difference. Dose-matched measurements and tissue-specific perturbation would be more informative than another undifferentiated association between total SCFAs and bone density.

#### 3.2.2 Bile acids

Bile acids are host–microbial co-metabolites: host synthesis and enterohepatic handling supply substrates that microbes subsequently transform. A circulating profile therefore cannot isolate microbial production. The farnesoid X receptor (FXR) and G protein-coupled bile acid receptor 1 (TGR5) have distinct distributions and effects, and some derivatives act through other targets. For example, 3-oxo-lithocholic acid and isoallo-lithocholic acid altered mouse Th17 or Treg differentiation through different mechanisms (24). This experiment establishes an upstream immune effect, but it did not measure a skeletal endpoint.

More direct skeletal evidence comes from an ageing-mouse study of hyodeoxycholic acid (HDCA) (25). HDCA inhibited osteoclast maturation, and TGR5 deletion attenuated its skeletal effects. Although *Parabacteroides goldsteinii* abundance tracked HDCA, transfer of this organism alone did not reproduce the benefit of young-donor microbiota. This dissociation is important: taxon association did not establish taxon sufficiency. The results support a defined preclinical HDCA–TGR5 route, but not all bile acids, FXR pathways or proposed T-cell mechanisms. Human exposure, safety and product-specific efficacy remain unestablished.

#### 3.2.3 Tryptophan derivatives

Tryptophan enters both microbial indole pathways and host metabolism, so an associated metabolite needs source attribution. In ovariectomized mice, indole-3-acetic acid (IAA) and indole-3-propionic acid improved barrier and bone measures (18). The study linked intestinal AhR activation to epithelial Wnt/β-catenin signaling and reported macrophage-associated interleukin-10 (IL-10) responses. Deleting AhR in intestinal epithelial cells markedly weakened the IAA response, identifying a requirement at the gut interface. The IL-10 observations do not establish transport of the cytokine from gut to marrow or prove that it mediates every skeletal effect. This distinction separates the experimentally tested epithelial requirement from the remaining immune mechanism.

### 3.3 Intestinal barrier function and systemic inflammation

An intact epithelial layer is only one component of barrier integrity. Colonocyte fuel use, mucus turnover, junctional proteins and local immune surveillance determine how closely luminal organisms approach host tissue. Butyrate can support several of these functions, but fecal butyrate abundance does not measure permeability in an individual patient.

A barrier-to-bone hypothesis contains several testable links: altered permeability, increased exposure to a specified microbial product, receptor engagement, a systemic or marrow response and skeletal change. LPS signaling through Toll-like receptor 4 (TLR4) and nuclear factor-κB (NF-κB) is one candidate route, not a generic description of every microbial ligand. Tight-junction protein abundance is not itself a permeability assay; circulating LPS is not evidence of viable bacterial translocation. Even simultaneous changes in these measures do not establish mediation. Sex-steroid-deficiency experiments and clinical co-measurement provide different parts of the argument (13, 15, 17). Where receptor blockade, mediator interruption or temporal sampling is absent, a continuous gut-to-bone mechanism remains an inference.

#### 3.3.1 Mineral handling and skeletal outcomes

Fermentation can lower luminal pH, change calcium solubility and alter epithelial transport, creating a credible mineral-handling component of the gut–bone axis. Short controlled trials found that soluble maize or corn fiber increased fractional calcium absorption in adolescents (26, 27). A one-year trial, however, did not show improved bone mineral content or density with soluble corn fiber and calcium (28). These studies concern growth rather than osteoporosis, but the contrast is informative. Improved absorption is a proximal physiological effect and does not establish durable skeletal benefit.

For immunological interpretation, a mineral effect also provides a competing explanation. A dietary intervention could alter BMD through calcium availability without changing marrow immunity. Conversely, a cytokine change could accompany improved nutrition without mediating bone benefit. Trials should prespecify which route is being tested and measure enough intermediate variables to distinguish these explanations.

### 3.4 Neuroendocrine regulation: the microbiota–gut–brain–bone axis

Neuroendocrine mechanisms broaden the gut–bone hypothesis but should remain secondary to better-tested immune and metabolic routes. Microbial regulation of host serotonin production has experimental support (29), while wider brain–gut interactions are discussed in established and recent reviews (8, 9, 30). Effects depend on the site of serotonin production and the responding tissue. A complete microbial exposure–neural mediator–skeletal outcome sequence has not been established by those observations alone. Osteoporosis-specific perturbation and measurement of intermediate signals are needed before this circuit can be assigned clinical importance.

### 3.5 Representative microbe–function relationships

Taxon-level associations vary across populations and analytic platforms. In 684 Chinese adults, shotgun metagenomics linked higher BMD with several Lachnospiraceae species and with L-arginine biosynthesis and butyrate-production pathways (31). The age-related decline in BMD was steeper in participants with a *Bacteroides* enterotype than in those with a *Prevotella* enterotype, and the pattern was assessed in an independent cohort. Broad differences in alpha or beta diversity were absent in the same study, which argues against a universal ‘low-diversity’ signature of osteoporosis.

Some postmenopausal cohorts report lower alpha diversity, fewer SCFA-producing taxa and enrichment of potential pathobionts. Other large datasets do not reproduce these global diversity changes (31). Associations with C-telopeptide, osteocalcin or hip BMD may identify candidate microbial features, but they are sensitive to age, diet, medication, geography, sequencing method and compositional analysis. Current signatures remain exploratory and have not been validated as clinical biomarkers.

Bacterial extracellular vesicles extend the candidate exposures beyond free metabolites. *Roseburia intestinalis*-derived vesicles promoted stromal-cell osteogenic readouts and improved bone measures in experimental osteoporosis (32). These findings concern a characterized experimental preparation, not a therapeutic property of every *Roseburia* strain. Translation requires reproducible product characterization and evidence that the relevant cargo reaches skeletal targets at an effective, safe exposure.

Functional profiling may bring associations closer to a testable exposure, but it is not inherently reproducible or causal. Different communities can encode the same pathway without expressing it at the same rate, and predicted functions have also failed to replicate between cohorts (33). We therefore treat community function as a candidate explanatory level, not an established universal signature. **Figure 1** distinguishes route-specific experimental support from the unconfirmed extensions to human marrow and clinical outcomes.

**Figure 1:**
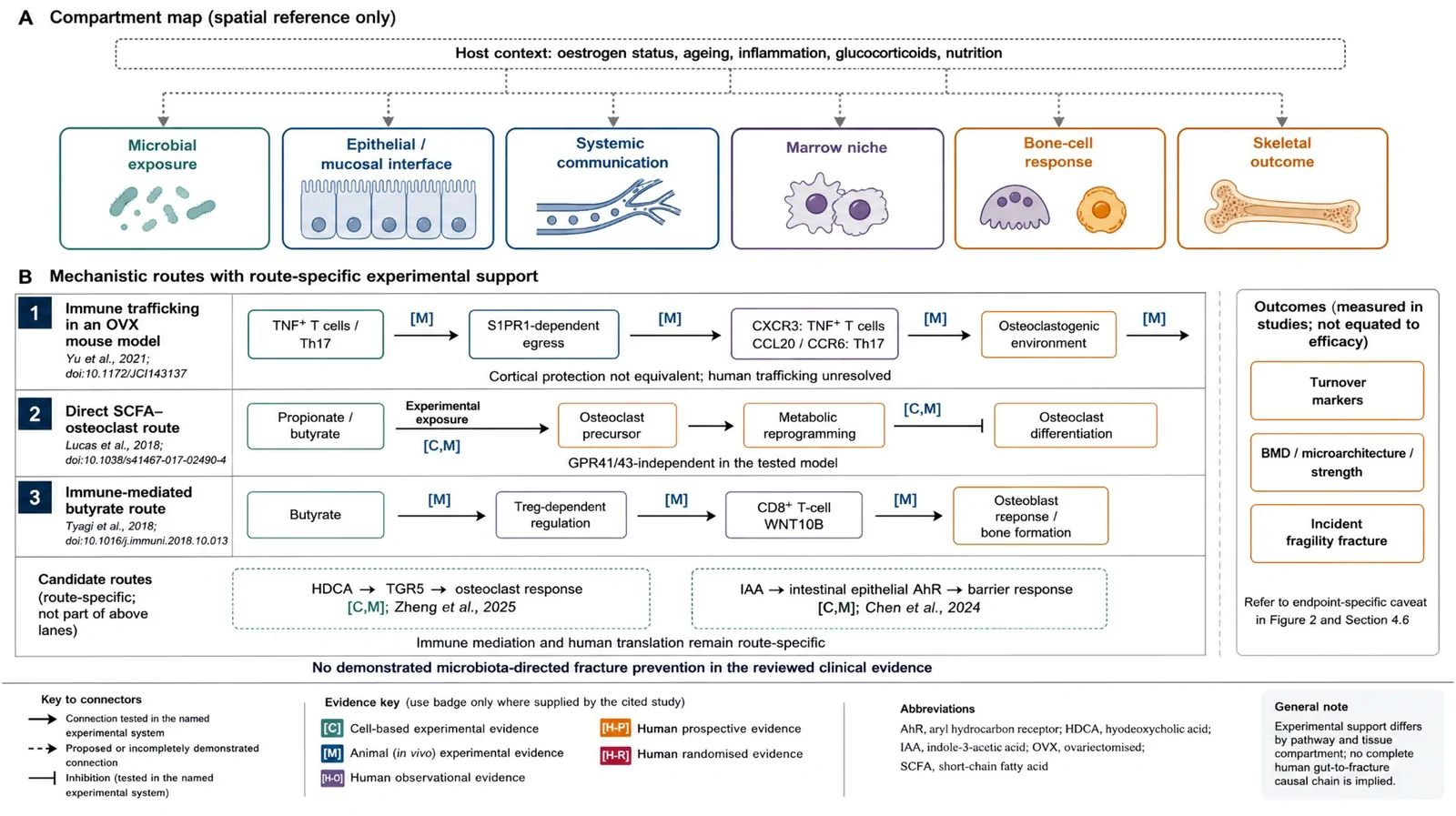
Route-specific evidence linking microbial exposures to skeletal responses. Panel A separates microbial exposure, epithelial/mucosal interface, systemic communication, marrow niche, bone-cell response and skeletal outcome, with host context modifying several compartments. Panel B distinguishes three experimentally studied routes: immune-cell trafficking in an ovariectomy model (16), propionate/butyrate effects on osteoclast metabolism (20), and Treg-dependent regulation of CD8+ T-cell WNT10B (21). These routes need not traverse identical compartments or require a microbial product to enter bone. Candidate HDCA–TGR5 and intestinal AhR routes are identified separately (18, 25). Solid arrows denote tested connections within the specified experimental system; dashed arrows denote proposed extensions, not negative findings. Evidence tags identify cell/animal or human designs. The arrow key indicates support for a connection, not treatment-effect magnitude. Bone-turnover markers, BMD/microarchitecture and incident fracture are kept distinct; no pathway is labeled as clinically proven fracture prevention. **Supplementary Material S2** provides the supporting studies and principal missing links.

## 4 Clinical evidence linking gut microbiota to osteoporosis

### 4.1 Microbial diversity and compositional alterations

Human studies report microbial differences across bone phenotypes, but these depend on sampling, sequencing, population and covariate control. Large primary cohorts do not establish a universal low-diversity signature (31, 33–35). Diversity is a community descriptor, not a direct assay of inflammatory function or a sufficient diagnostic test.

Relative abundance is a proportion: an apparent increase can arise because other community members decline. Sequencing depth is not a measurement of microbial biomass. A species-level association, a gene-content estimate, a transcript and a metabolite concentration describe different biological layers. Quantitative profiling that combines sequencing with cell counts illustrates how absolute microbial load can change ecological interpretation (36). This methodological example does not itself validate a bone biomarker. For each skeletal study, the assay actually performed must remain visible.

Larger cohorts sharpen this point. In two independent ageing cohorts totaling more than 2,000 participants, only a small subset of genus-level associations with volumetric density or microarchitecture replicated, and predicted functional pathways were not concordant between cohorts (33). A shotgun metagenomic study of 605 adults found no overall diversity difference across normal BMD, osteopenia and osteoporosis, but detected species-level associations that varied with polygenic risk (35). In 181 older adults, measured diet, medication and health variables explained a substantial share of community variation, while alpha diversity again showed no relation to BMD (34).

The sequencing-based entries in **Table 2** principally describe relative community features or gene content, not absolute bacterial counts or transcriptional activity. Shotgun metagenomics improves functional resolution but does not measure pathway flux. Where absolute load was not established, the review does not reinterpret an apparent enrichment as organism expansion. Calibration by flow cytometry, spike-in standards or other quantitative assays should be explicitly documented in future studies, rather than inferred from sequencing depth (36).

A genetically informed analysis of 1,104 taxa reported significant heritability for 96 and associations with estimated heel BMD (37). Signals involving Bifidobacteriaceae, *Bifidobacterium* and *Bifidobacterium adolescentis* were related to LCT/MCM6-region host architecture and metabolic pathways. This is not a trial of bifidobacterial supplementation. Genetic liability to a relative-abundance measure may involve diet, digestion or other host traits; it cannot be equated with administering a defined strain. The evidence is useful for hypothesis prioritization only to the extent that the instruments and biological interpretation are credible.

### 4.2 Metabolomic signatures

Metabolomics measures compounds rather than inferring them from microbial gene content. Associations with amino-acid, tryptophan and bile-acid pathways have been reported in low-BMD populations (38, 39). However, a circulating compound may derive from host metabolism, diet or microbial activity. Longitudinal sampling can establish temporal order but cannot, by itself, assign its source. Source attribution is stronger with isotope tracing or source-specific perturbation, supported by matched microbial functional measurements.

Integrated metagenomic and metabolomic analyses can connect community features with candidate effector molecules. In a peri- and postmenopausal cohort, *Bacteroides vulgatus* was linked to valeric-acid-related pathways and bone measures, followed by experimental validation (39). Other studies have proposed small microbial or metabolite panels for low bone mass. Clinical use would require blinded external validation, calibration across platforms and evidence of added value beyond age, BMD and established fracture-risk tools.

A community-based study of 1,776 adults linked osteoporosis with taxonomic and functional features and then used paired fecal and serum metabolomics to implicate amino-acid pathways (38). The design goes beyond listing organisms, but mediation inferred from cross-sectional data still needs temporal or experimental confirmation.

### 4.3 Inflammatory and immune parameters

Human immune evidence is still compartment-limited. The 77-woman study measured peripheral lymphocyte populations, serum cytokines and LPS alongside fecal microbiota (15). These measurements establish co-occurrence, not migration into marrow or a microbial cause of altered osteoclast function. Blood sampling can support feasible longitudinal studies, but a peripheral marker should not be labeled a marrow phenotype without validation. Stronger mediation claims would require appropriately timed exposure, immune and skeletal measurements together with an intervention or another credible identification strategy.

### 4.4 Causal inference and knowledge gaps

Confounding, reverse causation and mediation are distinct explanations for a microbiome–bone association. Diet, estrogen status or medication may affect both microbial features and bone without either causing the other. Conversely, osteoporosis, fracture or treatment can change activity, diet and gastrointestinal physiology, which may subsequently alter the microbiome. A mediator lies on the hypothesized causal route and should not automatically be adjusted away as a confounder. Selection can create another bias: if both frailty and gastrointestinal symptoms influence clinic attendance, restricting analysis to clinic attendees can induce a spurious association. Covariate selection therefore requires an explicit causal model, not the largest available adjustment set (40).

Mendelian randomization can reduce some confounding under valid instrumental-variable assumptions, but it does not automatically establish microbial causality (37). Weak instruments, horizontal pleiotropy, ancestry restriction and host loci affecting food consumption or digestion can undermine interpretation. Bidirectional analyses and sensitivity checks are informative but cannot guarantee that every violation has been excluded. If relevance, independence or exclusion restriction is doubtful, even an inferred direction of effect may be unreliable. Concordance with longitudinal measurements and exposure-specific experiments is needed before a genetic association becomes a therapeutic rationale.

Compositional analysis and confounder control address different problems. Negative laboratory controls, batch handling and harmonized metadata are needed alongside suitable relative-abundance analyses (40–42). Absolute load, measured metabolites and external validation can strengthen interpretation, but none alone removes confounding. **Table 2** identifies assay type rather than implying that sequencing directly measures production or exposure. STORMS offers a reporting framework for making these analytic decisions auditable (11).

### 4.5 Host context and effect modification

Host context can modify a microbial effect as well as confound its measurement. Estrogen deficiency affects epithelial and immune responses while acting directly on bone; inflammatory disease, glucocorticoids and frailty create different marrow and intestinal conditions. In CB6F1 mice, age-related bone loss occurred despite differences in germ-free status or colonization (43), whereas other models showed microbial dependence after sex-steroid withdrawal (13). The findings can coexist because they do not estimate the same effect in the same biological setting. Neither should be extrapolated to all osteoporosis phenotypes.

A host-context model therefore places hormonal state, inflammation, age, medication and nutrition upstream of several compartments, not only upstream of microbiome composition. The same microbial product may encounter different receptor expression, immune activation and skeletal turnover in different hosts. Clinical studies should define these contexts before analysis and distinguish effect modification from confounding. Geography, diet, antibiotics and stool transit still require harmonized measurement, but an extensive covariate list is not a substitute for a biologically justified model.

### 4.6 Clinical endpoints and evidence interpretation

Prospective data have moved the human literature beyond a catalog of cross-sectional taxa. In FINRISK, shotgun metagenomic profiles from 7,043 participants were related to 1,092 incident fractures during a median follow-up of 18 years (44). Associations involving diversity, Proteobacteria and functional pathways persisted after adjustment, which supports temporal relevance without establishing mediation. This prospective signal must be interpreted alongside inconsistent BMD signatures, model-dependent ageing effects (43) and mixed intervention trials. As **Figure 2** illustrates, target selection cannot rest on any one evidence layer.

**Figure 2:**
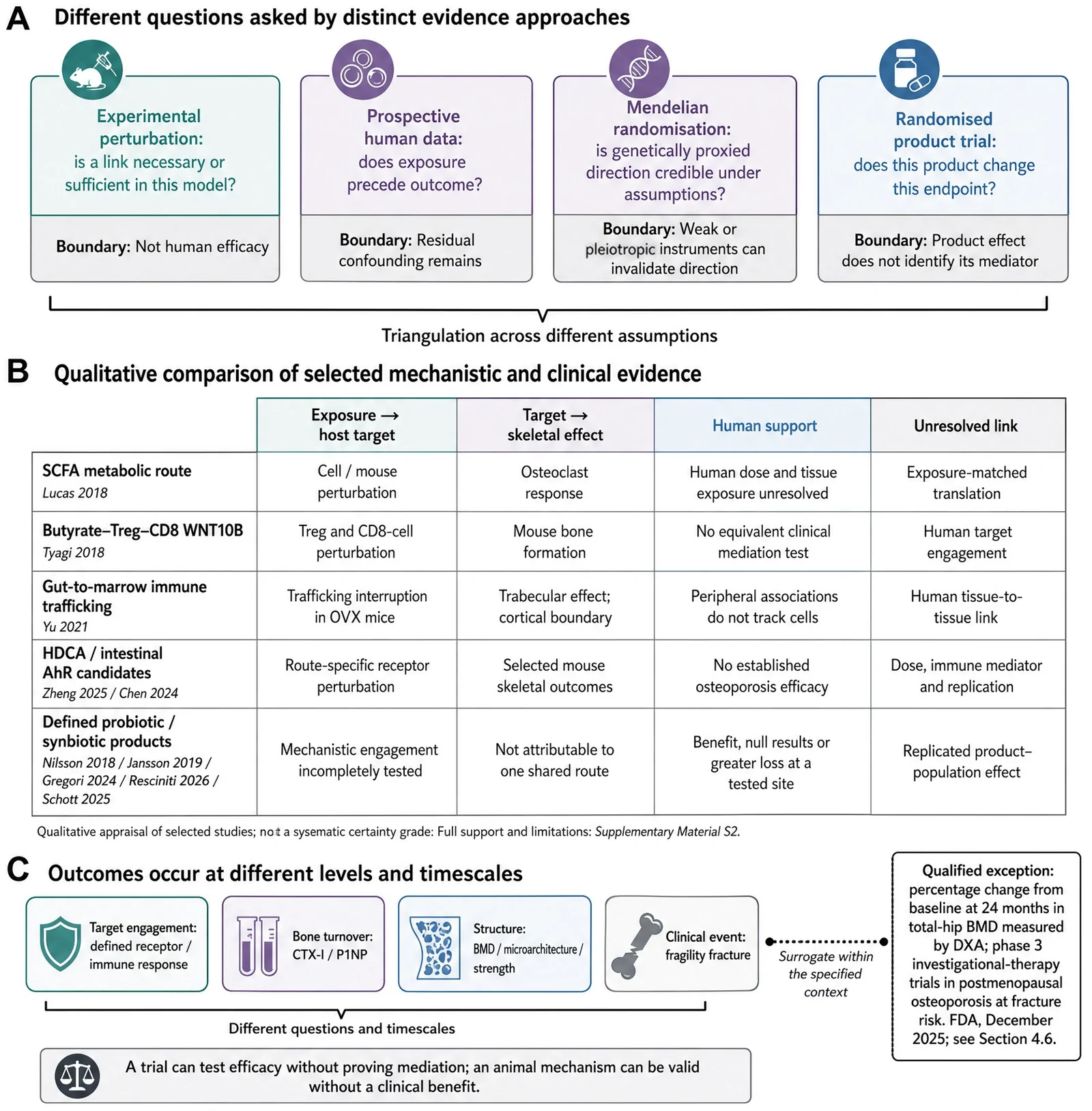
What complementary evidence can establish. Panel **(A)** contrasts experimental perturbation, prospective association, Mendelian randomization and randomized product testing. Panel **(B)** maps selected pathways to their measured support and unresolved links; it is an interpretive evidence map, not a formal certainty grade. Panel **(C)** distinguishes microbial target engagement, bone turnover, skeletal structure and fracture. These are not automatically interchangeable outcomes. A bounded inset notes the specific total-hip DXA endpoint qualified by the FDA, as described in Section 4.6 (45). Product efficacy can be established without identifying its mediator, whereas experimental mediation within a mouse model does not establish benefit in patients. Supporting studies and limits are provided in **Supplementary Material S2**.

Outcome interpretation requires four distinctions. Receptor activity and defined immune or metabolic responses can indicate microbial target engagement. Bone-turnover markers, including C-terminal telopeptide of type I collagen (CTX-I) and procollagen type I N-terminal propeptide (P1NP), measure downstream skeletal activity but are not specific to a microbial mechanism. Bone mineral density, microarchitecture and measured or estimated strength describe structural outcomes. Incident fragility fracture is a clinical event also influenced by falls, frailty and competing mortality. Thus, FINRISK’s prospective fracture associations (44) address a different question from a short-term change in a turnover marker.

Surrogacy is a context-specific qualification, not a blanket property of BMD. In December 2025, the US Food and Drug Administration (FDA) qualified percentage change in total-hip BMD measured by dual-energy X-ray absorptiometry (DXA) at 24 months as a fracture surrogate in phase 3 trials of investigational therapies for postmenopausal women with osteoporosis at fracture risk (45). This does not validate short-term spine or tibial changes, or turnover markers, as interchangeable endpoints. The microbiota trials summarized here should be interpreted according to their actual site, duration and population.

In many otherwise plausible causal chains, the mediator is the least well-supported link. Many cohorts infer fermentation or amino-acid metabolism from gene content without measuring the circulating product, while preclinical studies may administer exposures that are difficult to relate to humans. More informative studies would quantify microbial function, the candidate metabolite, host target engagement and the skeletal endpoint in the same participants. They would also test alternative sources, including diet-derived compounds and host metabolism, before assigning the pathway to the microbiota.

## 5 Microbiota-directed interventions

### 5.1 Probiotics

The evidence for probiotics is tied to the administered strain and preparation. Experimental reductions in bone loss have prompted clinical trials, but human results differ across products and skeletal sites. The pertinent clinical question is whether a specified preparation changes the prespecified endpoint in the intended population; a proposed immune mechanism requires an additional test.

Product identity is part of the mechanism. Strain accession, genomic verification, viability, dose, storage stability, accompanying substrate and preparation method determine what the host receives. Persistence should be measured rather than inferred from ingestion, and permanent colonization need not be required for a transient product effect. A strain-associated immune change is not transferable to every strain of the same species. Mouse experiments can nominate candidates (55), but clinical testing should connect the administered material to a specified immune or metabolic target as well as a skeletal endpoint.

Lactic acid bacteria have received the most clinical attention. *Limosilactobacillus reuteri* ATCC PTA 6475, reported under its former genus name in the original publication, reduced tibial volumetric BMD loss over 12 months in older women with low BMD (46). A larger two-year trial in early postmenopausal women did not detect benefit at the tibia, spine or hip (48). Differences in age, baseline bone loss and treatment duration may have contributed, but the null result also limits claims of a class-wide osteoprotective effect.

*Lacticaseibacillus rhamnosus* GG reduced bone loss in sex-steroid-deficient mice by limiting permeability and inflammatory signaling (13). Other defined probiotic preparations have protected cortical bone in ovariectomized mice (55). The active route may involve short-chain fatty acids, Treg responses and RANKL/OPG regulation, but the measured mechanism and skeletal compartment differ across models.

*Bifidobacterium animalis* subsp. *lactis* A6 attenuated bone and muscle loss in mouse models of antibiotic- or dextran-sulfate-associated gut dysfunction, partly through recovery of butyrate production and lower NF-κB activity (56). These models clarify how intestinal injury can influence musculoskeletal tissues. They are not direct models of uncomplicated postmenopausal osteoporosis, and human efficacy remains unknown.

Clinical findings remain mixed even when strain accessions match. Jansson et al. reported less lumbar-spine bone loss with a three-strain preparation (47), whereas Resciniti et al. found greater distal-tibial loss with the same accessions and daily viable count (49). The primary sites and measurement methods differed, so this is neither an exact replication nor proof that a previously beneficial formulation became harmful. It does show why strain identity alone cannot predict a skeletal result. Population, formulation and endpoint must be considered together. SBD111 likewise yielded a null primary result in non-osteoporotic early postmenopausal women (50). Earlier combination and multispecies trials add evidence but differ in co-interventions and outcomes (57–59).

The small CCFM1126 study needs a different qualification (51). Forty-four participants entered the study and 31 remained after withdrawals; the report’s allocation and attrition chronology are not fully clear. The human preparation supplied 10^10^ colony-forming units (CFU) per day, distinct from doses used in its animal experiments. Changes in DXA-derived T- and Z-scores favored treatment, but the skeletal site was not specified in the BMD assessment method and a standard absolute BMD effect with confidence interval was not supplied there. Most reported serum changes also lacked a significant between-group contrast. These results warrant replication with clearer outcome and analysis-population reporting before being used to support immune mediation or clinical benefit.

Published meta-analyses report site-specific BMD and turnover-marker effects across heterogeneous products and populations (52–54, 60, 61). Several reuse the same trials, so agreement between pooled estimates is not independent replication. Their search dates also determine whether recent null or discordant trials contribute. **Table 2** retains the primary studies alongside the syntheses to make this distinction visible. RANKL effects are clearer in animal experiments than in the limited human trials (19); pooling an immune readout cannot supply the missing clinical mechanism.

*Akkermansia muciniphila* illustrates the problem of assigning a therapeutic property to a species name. Its relevance depends on strain, preparation and host ecology, while associations with BMD do not establish a treatment effect. A live organism, a pasteurized preparation, a purified component and a vesicle formulation are different exposures. Evidence for engineered vesicle cargo should not be reinterpreted as evidence that the native organism supplies the same cargo at an effective dose (62).

The International Scientific Association for Probiotics and Prebiotics (ISAPP) defines a postbiotic as a beneficial preparation of inanimate microorganisms and/or their components (63). Purified metabolites are a different category. Heat inactivation establishes a processing step, not benefit, and the preparation cannot inherit evidence from a live strain. Comparator choice should follow the claim: a live preparation may warrant an inactivated comparator, while engineered vesicles require matched empty-vesicle and free-cargo controls.

For native and engineered bacterial extracellular vesicles (BEVs), cargo identity, dose, route and distribution require separate evaluation (32, 62). An engineered preparation that affects mouse bone may deliver a different exposure from naturally released intestinal vesicles. Immune stimulation and off-target uptake also depend on cargo and preparation quality. Evidence of bone activity should therefore be accompanied by quantitative biodistribution and appropriate controls, rather than an assumption that microbial origin confers targeting or safety.

### 5.2 Prebiotics and synbiotics

A prebiotic is a substrate selectively utilized by host microorganisms that confers a health benefit (64). This definition is more precise than the older description of a non-digestible ingredient that stimulates selected ‘beneficial’ taxa, and it covers a broader range of substrates. Inulin-type fructans, galacto-oligosaccharides and some fermentable fibers can alter community function and SCFA output, but a compositional shift alone does not demonstrate skeletal benefit.

Prebiotic studies should establish utilization of the administered substrate and relate the resulting exposure to the intended host response. Calcium absorption is one possible intermediate, as discussed in Section 3.3.1 (26–28). Where a skeletal effect is attributed to microbial metabolism rather than nutrition alone, a matched substrate or nutritional comparator and repeated mediator measurements would help distinguish those explanations.

In the SBD111 trial, 286 non-osteoporotic early postmenopausal women received the specified synbiotic or placebo (50). The primary lumbar-spine result was null. Subgroup findings in women with particular body mass index or osteopenia profiles warrant prospective testing, with a treatment-by-subgroup interaction analysis; a significant estimate within one subgroup is insufficient. **Table 2** supplies the principal effect estimate, and **Supplementary Material S2** records the organism and substrate doses.

### 5.3 Dietary interventions

Diet changes microbial substrate availability and metabolic output, but it also acts directly on bone through energy balance, protein and mineral intake, vitamin status and body composition. Attribution to the microbiota requires measurement of the proposed microbial mediator and evidence that it precedes the skeletal response.

Controlled feeding can reduce variation in nutrient intake, while repeated microbiome and host measurements can establish whether a nominated mediator precedes skeletal change. Neither approach alone resolves mediation, but together they allow a more informative test than a post-intervention association. This distinction is particularly important when diet is changed together with exercise or weight loss.

Dietary interventions remain clinically attractive because they can accompany established osteoporosis care. Their evaluation should distinguish an ecological exposure from a defined microbial product and prespecify whether the intended outcome is target engagement, mineral absorption, BMD or fracture. That separation would prevent a short-term physiological effect from being presented as durable osteoprotection.

A three-year secondary analysis of PREDIMED-Plus found that an energy-reduced Mediterranean diet combined with physical activity attenuated BMD decline in older women with metabolic syndrome (65). Because the intervention changed energy intake, weight and exercise as well as diet, the result cannot be assigned specifically to the microbiota. It nevertheless provides a clinically realistic context in which microbial mediation could be tested.

### 5.4 Fecal microbiota transplantation

Fecal microbiota transplantation (FMT) transfers a complex community, not a defined strain. This broad ecological perturbation is informative in causal animal experiments, but it creates substantial problems of safety, reproducibility and mechanistic attribution.

Control-donor FMT improved bone microarchitecture, junctional proteins and microbial metabolic profiles in ovariectomized mice (17). The experiment supports a community-level intervention effect but does not identify a necessary organism, metabolite or immune-cell mediator. Other transfer studies report bone loss after exposure to communities associated with estrogen deficiency (66). These results should not be compressed into a universal beneficial or harmful FMT mechanism.

FMT can transfer organisms, phages, metabolites and mobile genetic elements, including antimicrobial-resistance determinants (67, 68). Engraftment depends on the donor–recipient ecological match, prior antibiotics, diet and host immunity. Transfer from women with postmenopausal osteoporosis accelerated bone loss in antibiotic-treated mice, but only five donors per group were used (69). Multiple recipient mice do not create additional independent human donors. Donor-level replication and appropriate clustering are therefore important. Reconstructing a defined consortium, removing a candidate function and rescuing the phenotype would test a mediator more directly than whole-community transfer alone.

Immune consequences extend beyond infection. A transferred community may encounter a different mucosal barrier, antigenic history and inflammatory state, changing tolerance or inflammatory signaling. Such possibilities warrant measurement rather than an assumption that donor microbiota is intrinsically healthy or osteoprotective. No controlled human trial in the evidence reviewed establishes FMT efficacy for osteoporosis. Guidance on clinical FMT use stresses defined indications, donor screening, manufacturing and recipient risk (67); it does not establish an osteoporosis indication.

The safety concern is supported by clinical experience. Transmission of an extended-spectrum beta-lactamase-producing *Escherichia coli* through investigational FMT caused invasive infections in immunocompromised recipients, including one death (68). Because osteoporosis is common in older adults and in patients exposed to immunosuppressive treatment, use of an undefined microbial community would require an unusually strong indication and rigorous risk controls.

### 5.5 Emerging strategies: engineered probiotics and advanced delivery systems

Engineered probiotics and bacterial extracellular vesicles are being developed as delivery systems for metabolites, nucleic acids and proteins. These platforms can improve stability or tissue targeting in animals, but engineering also changes dose, biodistribution and immunological exposure. Descriptions such as biocompatible or low toxicity are premature without appropriate comparative and long-term testing.

In 2026, bone-targeted *A. muciniphila* vesicles carrying miR-21-5p improved bone measures in ovariectomized mice through phosphoinositide 3-kinase (PI3K)–protein kinase B (AKT) signaling (62). Defining the cargo and perturbing the proposed pathway makes the preparation experimentally informative. Whether native vesicles deliver a comparable exposure, and whether this engineered product has an acceptable human safety profile, are separate questions.

*Roseburia intestinalis*-derived vesicles were linked to histidylleucine and AKT/forkhead box O1 (FOXO1)-related osteogenic responses in cells and an animal osteoporosis model (32). Administration of a candidate metabolite can demonstrate sufficiency under the tested conditions, but does not by itself establish that it is necessary for every vesicle effect. Independent replication, mediator-specific interruption and quantitative exposure studies are needed to establish the contribution of this route to the response of the intact preparation.

These interventions differ in biological scope. Diet, prebiotics and FMT perturb an ecosystem; defined strains alter a narrower exposure; inactivated preparations retain selected structures; metabolites and engineered cargo test particular molecules. Their immune actions need not converge on one pathway. A product should be classified by the material administered and the target response demonstrated, not solely by its microbial origin. **Table 3** keeps these categories separate.

**Table 3:** Product-specific evidence gaps and development requirements.

Intervention class	What is actually administered	Evidence and main uncertainty	Next discriminating test
Diet/prebiotic	Dietary pattern or defined selectively utilised substrate (64, 65)	Physiological and lifestyle evidence; bone effects may be nutritional rather than microbial	Separate nutrient/activity effects from measured microbial mediation
Defined live strain	Genomically specified viable organism, dose and shelf-life potency	Mixed product-specific RCT results (46–49, 51)	Replicate identity, exposure and prespecified skeletal response in the target population
Synbiotic	Specified organism(s) and substrate(s)	SBD111 primary endpoint null in non-osteoporotic women (50)	Prospective phenotype selection and verified host target engagement
Inactivated preparation/postbiotic	Inanimate microorganisms and/or components with demonstrated benefit; not simply a purified metabolite (63)	Preparation-specific properties; cannot inherit live-strain efficacy	Compare active preparation with live and matched process controls; define potency
Purified metabolite	Specified molecule, formulation and route	Preclinical SCFA, HDCA and indole evidence (18, 20, 25); achievable human exposure uncertain	Pharmacokinetics, receptor/tissue response and exposure–response window
Engineered live strain	Modified organism with verified construct and output	An engineering strategy, not an established osteoporosis therapy	Genetic stability, containment, off-target function and dose control
Native/engineered BEV	Vesicle preparation with quantified cargo, purity and route	Cell and animal proof of concept (32, 62); immunogenicity and natural human exposure uncertain	Empty-vesicle/free-cargo controls, biodistribution, potency and repeat-dose safety
FMT	Donor-derived ecosystem with accompanying phages, metabolites and mobile elements	Model-specific transfer effects (17, 66, 69); no established human osteoporosis efficacy	Donor-level replication and defined-function dissection; regulated safety evaluation before clinical development

### 5.6 Product-specific development requirements

Clinical readiness depends on product identity, achievable exposure, target engagement, manufacturing, safety and an appropriate endpoint. Better compositional definition does not itself establish efficacy, and broad ecological effects may not be reproduced by a purified molecule. **Table 3** and **Figure 3** present development requirements without assigning numerical rankings or implying that any microbiota-directed product is an established osteoporosis treatment.

**Figure 3:**
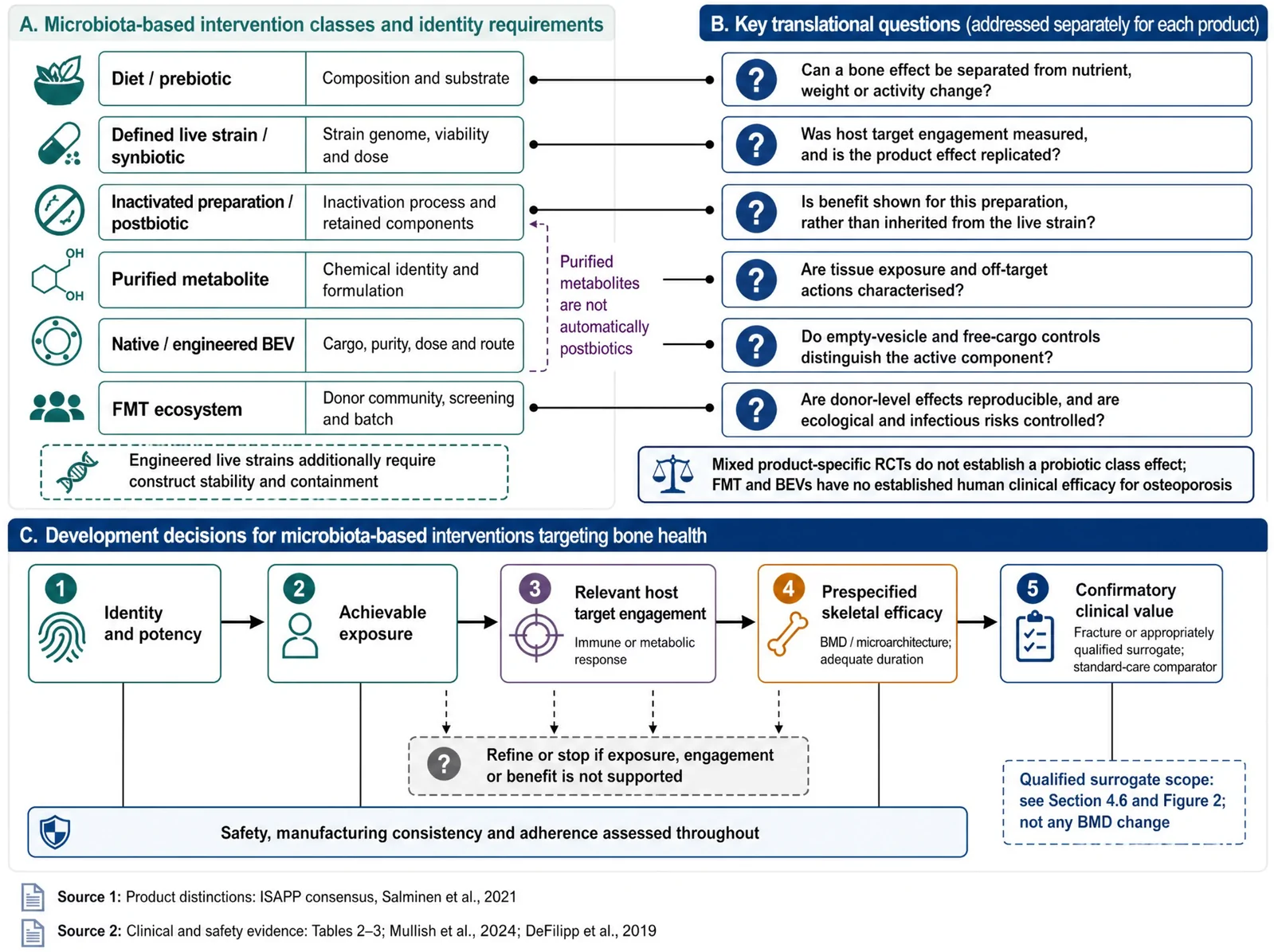
Product-specific development decisions. Panels **(A, B)** distinguish ecological interventions, defined live products, inactivated preparations, purified metabolites and vesicle systems, with the corresponding identity, exposure and safety questions. Panel **(C)** follows development from a defined preparation to host target engagement and prespecified skeletal testing. It distinguishes biological proof of mechanism from confirmatory clinical assessment, including an appropriately qualified surrogate where applicable. Process arrows describe decisions, not established causal links or approval. Inconclusive or negative evidence prompts refinement or stopping; it does not automatically justify progression. **Tables 2**, **3** provide the clinical context.

Product reporting should describe the administered material and its potency at use, including storage and adherence. For the three-strain trials, matching accession numbers and daily counts still leaves differences in manufacturing, host ecology and endpoint ascertainment to examine (47, 49). A metabolite instead requires exposure–response characterization, and a vesicle preparation requires cargo and purity controls. **Table 3** links these distinct questions to tests that could resolve them.

Comparator choice determines clinical relevance. A microbiota-directed product should be evaluated alongside appropriate osteoporosis care, with sufficient follow-up for the chosen endpoint. The endpoint must support the intended claim: mechanistic activity, change in bone turnover and clinically meaningful skeletal benefit require different interpretations. Section 4.6 explains the limited context in which an imaging endpoint can support a surrogate claim. It does not justify relabeling every BMD change as fracture prevention.

## 6 Discussion: controversies, methodological priorities, and translational boundaries

### 6.1 Why the literature does not yet converge

Several kinds of disagreement need to be separated. Taxonomic inconsistency may reflect compositional measurement and analytic choices (40–42). By contrast, divergent responses to a specified product raise questions about dose, host physiology and outcome. The three-strain trials illustrate the latter problem: a spine signal and a tibial signal cannot be treated as estimates of one identical endpoint (47, 49). Such distinctions matter before invoking a general failure of microbiome reproducibility.

Apparent mechanistic conflicts can also identify boundaries rather than errors. Microbial dependence after sex-steroid withdrawal (13) and microbiome-independent ageing loss in another mouse model (43) need not be incompatible. Similarly, a receptor-independent osteoclast response to propionate or butyrate (20) can coexist with receptor-related acetate effects on marrow-cell differentiation (23). The relevant comparison is between specified exposures and biological settings, not between broad labels such as ‘SCFAs’ or ‘dysbiosis’.

The host-context interpretation is testable, but remains a hypothesis. If hormonal or inflammatory state changes the response to a fixed microbial exposure, a prospectively defined interaction should be reproducible while product identity and endpoint remain constant. An alternative is that apparent heterogeneity arises from measurement, attrition or chance. Current cross-study contrasts cannot distinguish these explanations. This is why retrospective responder narratives should lead to a new test rather than a clinical recommendation.

### 6.2 Methodological priorities

Longitudinal studies should link strain-resolved profiles to measured metabolites and host responses in the same participants, with sampling before changes in disease or treatment. Absolute microbial quantification and appropriate compositional analysis answer related but different questions. A predeclared causal model should guide adjustment, because medication or nutritional status can act as a confounder, mediator or consequence of disease depending on when it is measured. External replication should preserve the exposure definition and skeletal endpoint.

Mendelian randomization can help prioritize pathways when instruments are biologically interpretable and assumptions remain credible (37). Weak instruments and pleiotropic host loci can undermine both causal and directional claims. Concordance across genetic, longitudinal and experimental evidence is most useful when the designs have different plausible biases, not merely when they repeat the same association. Neither a sensitivity analysis nor a favorable genetic estimate replaces direct testing of a defined exposure and mediator.

Clinical stratification should use a small number of biologically justified variables specified before treatment. Interaction analyses need adequate power; nominal significance within one subgroup does not demonstrate that groups respond differently. Any microbiome-derived predictor should be fixed before evaluation in an independent population. Otherwise, feature selection and response assessment can reuse the same data and exaggerate performance.

Mechanistic studies should distinguish the experimental unit from repeated measurements. Separate donor preparations, cages or independently produced vesicle batches may constrain replication even when many mice, wells or sequencing reads are available. FMT and vesicle experiments are particularly vulnerable to treating recipients or technical replicates as independent evidence for a donor- or batch-level claim. Randomization and analysis should match the level at which the exposure was assigned.

Clinical reports should retain assigned-group analyses, account for missing outcomes and describe adherence without excluding non-adherent participants solely to improve the estimate. Repeated within-group tests do not establish a treatment effect; the comparison must address the between-group contrast with uncertainty. The same discipline applies to proposed mediators. Parallel improvement in an immune marker and BMD is insufficient unless the design supports the claim that changing that marker contributed to the skeletal response.

Trial duration and precision should match the claim. Imaging quality assurance and measurement error matter, but an individual patient’s least significant change should not be used as a universal threshold for a group-average trial effect. Multiplicity must be addressed when several skeletal sites or biomarkers are tested. Safety assessment should cover infection and metabolic or treatment interactions as appropriate to the product, alongside gastrointestinal tolerability.

### 6.3 Translational boundaries and a staged research agenda

Two comparisons offer concrete starting points. Trials using matched *L. reuteri* accessions differed in participant age and duration (46, 48), while three-strain trials with matching accessions and daily counts differed in their primary skeletal sites (47, 49). A replication program should hold the product and endpoint constant before testing hormonal or inflammatory modifiers. If a candidate phenotype is proposed, the interaction and direction of response should be specified in advance. Cross-study differences alone cannot establish that phenotype.

Experimental development can then ask whether a proposed mediator is necessary. Function-depleted consortia, receptor interruption or mediator depletion, followed by a suitable rescue, would distinguish a nominated pathway from broader community effects. Clinical development requires a related but separate decision: was the exposure achieved, did the intended host response occur and was the prespecified skeletal effect supported? A sufficiently precise result excluding a prespecified meaningful skeletal benefit, despite verified exposure and engagement, weighs against clinical relevance in that setting. An imprecise non-significant estimate remains inconclusive. Failure to achieve exposure instead points to delivery or adherence. Both outcomes are more informative than selecting a favorable secondary finding after the primary analysis fails.

### 6.4 Evidence boundaries

Established bone biology includes RANKL/RANK/OPG regulation of osteoclast development (3, 4). It does not establish a microbial treatment. Stronger preclinical support concerns specific links tested by perturbation, such as SCFA effects on osteoclast metabolism, Treg regulation of CD8+ T-cell WNT10B and immune trafficking in an ovariectomy model (16, 20–22). These conclusions retain their model and tissue boundaries.

Human evidence is suggestive but heterogeneous: prospective fracture associations and randomized product effects answer different questions (44, 46–50). Positive trial results merit the same attention as null or unfavorable results, while each retains its endpoint and analysis limitations. A portable microbial biomarker, several proposed human marrow exposures and a clinically effective microbial mechanism remain unresolved. These categories are an interpretive synthesis, not formal certainty grades; **Supplementary Material S2** records the selected evidence and the missing tests.

## 7 Conclusion

Microbial exposures influence bone in defined experimental systems. SCFA effects on osteoclast metabolism, Treg-regulated CD8+ T-cell WNT10B and intestinal immune trafficking provide testable examples (16, 20, 21). The strength of these experiments lies in perturbing particular links, not in completing a universal gut-to-bone diagram. Bile-acid, indole and vesicle studies extend the candidate mechanisms but require equally careful attribution of exposure, responding tissue and mediator (18, 25, 32, 62).

Human evidence remains product- and endpoint-specific. Trials include favorable findings, null primary results and greater bone loss at a tested site. The clinical evidence reviewed is insufficient to establish that microbiota-directed interventions reduce fragility-fracture risk. The appropriate response is to retain these contrasts and test their explanations, using clinically justified endpoints and effective standard care (1, 2). A reproducible microbial function may ultimately prove more useful than a taxonomic label, but that advantage still requires demonstration.

Progress will depend on resolving a few consequential uncertainties: which hosts respond, whether the intended exposure reaches its target, and whether that response improves a prespecified skeletal outcome. The evidence map identifies experiments capable of changing these judgements. It also distinguishes the limits of narrative evidence selection from the biological gaps that still separate a proposed mechanism from a treatment.
