# Supplementary material for: The gut–bone axis in osteoporosis: microbial mechanisms, conflicting evidence, and translational priorities
Source: Front Immunol. 2026 Oct 14;17:1966537. doi: 10.3389/fimmu.2026.1966537 (PMC13714726; doi:10.3389/fimmu.2026.1966537)
Supplement: Supplementary file 1 [file DataSheet1.docx]

Supplementary Search Methods and Evidence Appraisal

Manuscript 1966537 | Revised 20 September 2026

S1 describes literature identification, technical refinements to the search strategy and the roles of direct and contextual evidence. S2 presents the selected-study appraisal accompanying the review.

# S1 Literature search and source selection

## S1.1 Search chronology and responsibilities

| **Item** | **Search record** |
| --- | --- |
| Initial search | 1 December 2025 |
| Database update | 20 July 2026 |
| Coverage | Available database records through the July search; foundational studies were not excluded by a recent-year lower limit. |
| Databases and platforms | PubMed including MEDLINE (NCBI); Web of Science Core Collection (Clarivate); Scopus (Elsevier); Cochrane Library (Wiley). |
| Search roles | Zhe Liu conducted the searches; Chunli Yu reviewed the search strategy. |
| Detailed appraisal | English-language full texts. Language eligibility and access to full text are separate criteria; free-full-text availability is not an eligibility requirement. |
| Revision-stage checks | Reference-led checks on 19–20 September 2026 using Crossref, PubMed/Europe PMC and publisher or official records. |

The July date identifies the database update, not the final date of every source checked during revision. Revision-stage additions were assessed against the same substantive evidence boundaries. The initial date is specified in the author-supplied search record; the submitted manuscript reported it at month-level precision.

## S1.2 Search architecture and technical amendments

The core search combines intestinal-microbiota concepts with skeletal concepts. A separate product and mediator search retains studies that name an organism, preparation or metabolite without using a general microbiota term. Upstream searches address immune, epithelial, endocrine and mineral-absorption mechanisms. Findings from those searches require assessment of microbial relevance and are not automatically direct skeletal evidence.

The formulations in S1.3 are technical revisions of the supplied search record, not verbatim copies of its earlier strings. They align the two-domain core with Section 2.2 and make the supplementary searches explicit. They do not assign new retrieval or screening counts to these amendments.

| **Element** | **Technical refinement and rationale** |
| --- | --- |
| Core combination | Use microbiota AND skeleton, rather than requiring a third mechanism/intervention domain. A nested three-domain search is a focused subset, not an additional source of records. |
| Microbial specificity | Retain Gastrointestinal Microbiome; do not let the anatomical Gastrointestinal Tract heading alone satisfy microbial exposure. Pair generic profiling terms with intestinal context. |
| Skeletal coverage | Add bone mass, mineral content, osteopenia, microarchitecture and strength; include British and American remodelling spellings. |
| Product and mediator coverage | Add bacterial vesicles, indoles and receptor terms; cover faecal/fecal and transplantation variants. Include selected product identifiers and old/new bacterial names relevant to the review. |
| Source roles | Separate direct skeletal evidence from upstream biological context, background literature, reporting guidance and evidence syntheses. |

## S1.3 Database-specific formulations

Numbered sets must be created in the stated order within a search session. Scopus strings are supplied in full. The strings use standard straight quotation marks. Core and product/mediator results overlap and require deduplication; upstream results remain a separate source category. No numerical result is inferred from the number of citations in the manuscript.

### S1.3.1 PubMed including MEDLINE

MeSH terms are combined with title/abstract terms. The free-text fields make term behaviour explicit rather than relying solely on automatic mapping.

**#1 Microbiota**

"Gastrointestinal Microbiome"[MeSH Terms] OR ((gut[tiab] OR intestin*[tiab] OR gastrointestinal[tiab] OR faecal[tiab] OR fecal[tiab] OR colonic[tiab] OR caecal[tiab] OR cecal[tiab]) AND (microbiot*[tiab] OR microbiom*[tiab] OR microflora[tiab] OR dysbios*[tiab] OR metagenom*[tiab] OR "16S rRNA"[tiab])) OR "gut-bone axis"[tiab] OR "gut bone axis"[tiab]

**#2 Skeleton**

"Osteoporosis"[MeSH Terms] OR "Bone Density"[MeSH Terms] OR "Bone Remodeling"[MeSH Terms] OR "Fractures, Bone"[MeSH Terms] OR "Osteoclasts"[MeSH Terms] OR "Osteoblasts"[MeSH Terms] OR osteoporos*[tiab] OR osteopeni*[tiab] OR "bone mineral density"[tiab] OR BMD[tiab] OR "bone mineral content"[tiab] OR "bone mass"[tiab] OR "bone loss"[tiab] OR "bone resorption"[tiab] OR "bone formation"[tiab] OR "bone turnover"[tiab] OR "bone remodelling"[tiab] OR "bone remodeling"[tiab] OR "bone microarchitecture"[tiab] OR "bone strength"[tiab] OR fracture*[tiab] OR osteoclast*[tiab] OR osteoblast*[tiab] OR osteocyt*[tiab]

**#3 Products and mediators**

"Probiotics"[MeSH Terms] OR "Prebiotics"[MeSH Terms] OR "Synbiotics"[MeSH Terms] OR "Fecal Microbiota Transplantation"[MeSH Terms] OR "Fatty Acids, Volatile"[MeSH Terms] OR "Bile Acids and Salts"[MeSH Terms] OR probiotic*[tiab] OR prebiotic*[tiab] OR synbiotic*[tiab] OR postbiotic*[tiab] OR "faecal microbiota transplant*"[tiab] OR "fecal microbiota transplant*"[tiab] OR FMT[tiab] OR "Lactobacillus reuteri"[tiab] OR "Limosilactobacillus reuteri"[tiab] OR "Lactobacillus plantarum"[tiab] OR "Lactiplantibacillus plantarum"[tiab] OR "Lactobacillus paracasei"[tiab] OR "Lacticaseibacillus paracasei"[tiab] OR "Lactobacillus fermentum"[tiab] OR "Limosilactobacillus fermentum"[tiab] OR Bifidobacterium[tiab] OR "Bacillus subtilis"[tiab] OR "Akkermansia muciniphila"[tiab] OR "Roseburia intestinalis"[tiab] OR SBD111[tiab] OR CCFM1126[tiab] OR "short-chain fatty acid*"[tiab] OR "short chain fatty acid*"[tiab] OR SCFA*[tiab] OR butyrate[tiab] OR propionate[tiab] OR acetate[tiab] OR "bile acid*"[tiab] OR "hyodeoxycholic acid"[tiab] OR TGR5[tiab] OR FXR[tiab] OR tryptophan[tiab] OR indole*[tiab] OR "aryl hydrocarbon receptor"[tiab] OR AhR[tiab] OR "bacterial extracellular vesicle*"[tiab] OR "bacterial membrane vesicle*"[tiab] OR "outer membrane vesicle*"[tiab]

**#4 Upstream context**

"calcium absorption"[tiab] OR "mineral absorption"[tiab] OR "intestinal permeability"[tiab] OR "epithelial barrier"[tiab] OR lipopolysaccharide*[tiab] OR TLR4[tiab] OR Th17[tiab] OR Treg*[tiab] OR "regulatory T cell*"[tiab] OR "regulatory T-cell*"[tiab] OR RANKL[tiab] OR osteoprotegerin[tiab] OR WNT10B[tiab] OR serotonin[tiab] OR "insulin-like growth factor"[tiab] OR "IGF-1"[tiab]

**#5 Core search**

#1 AND #2

**#6 Product and mediator supplement**

#2 AND #3

**#7 Upstream context supplement**

#1 AND #4

### S1.3.2 Web of Science Core Collection

TS searches topic fields. The supplied record lists SCI-EXPANDED, SSCI, CPCI-S and CPCI-SSH; those selections are distinct from the query itself.

**#1 Microbiota**

TS=(((gut OR intestin* OR gastrointestinal OR faecal OR fecal OR colonic OR caecal OR cecal) AND (microbiot* OR microbiom* OR microflora OR dysbios* OR metagenom* OR "16S rRNA")) OR "gut-bone axis" OR "gut bone axis")

**#2 Skeleton**

TS=(osteoporos* OR osteopeni* OR "bone mineral density" OR BMD OR "bone mineral content" OR "bone mass" OR "bone loss" OR "bone resorption" OR "bone formation" OR "bone turnover" OR "bone remodelling" OR "bone remodeling" OR "bone microarchitecture" OR "bone strength" OR fracture* OR osteoclast* OR osteoblast* OR osteocyt*)

**#3 Products and mediators**

TS=(probiotic* OR prebiotic* OR synbiotic* OR postbiotic* OR "faecal microbiota transplant*" OR "fecal microbiota transplant*" OR FMT OR "Lactobacillus reuteri" OR "Limosilactobacillus reuteri" OR "Lactobacillus plantarum" OR "Lactiplantibacillus plantarum" OR "Lactobacillus paracasei" OR "Lacticaseibacillus paracasei" OR "Lactobacillus fermentum" OR "Limosilactobacillus fermentum" OR Bifidobacterium OR "Bacillus subtilis" OR "Akkermansia muciniphila" OR "Roseburia intestinalis" OR SBD111 OR CCFM1126 OR "short-chain fatty acid*" OR "short chain fatty acid*" OR SCFA* OR butyrate OR propionate OR acetate OR "bile acid*" OR "hyodeoxycholic acid" OR TGR5 OR FXR OR tryptophan OR indole* OR "aryl hydrocarbon receptor" OR AhR OR "bacterial extracellular vesicle*" OR "bacterial membrane vesicle*" OR "outer membrane vesicle*")

**#4 Upstream context**

TS=("calcium absorption" OR "mineral absorption" OR "intestinal permeability" OR "epithelial barrier" OR lipopolysaccharide* OR TLR4 OR Th17 OR Treg* OR "regulatory T cell*" OR "regulatory T-cell*" OR RANKL OR osteoprotegerin OR WNT10B OR serotonin OR "insulin-like growth factor" OR "IGF-1")

**#5 Core search**

#1 AND #2

**#6 Product and mediator supplement**

#2 AND #3

**#7 Upstream context supplement**

#1 AND #4

### S1.3.3 Scopus

TITLE-ABS-KEY searches title, abstract and keyword fields. No broad subject-area restriction is added to these cross-disciplinary query blocks.

**Core search**

TITLE-ABS-KEY(((gut OR intestin* OR gastrointestinal OR faecal OR fecal OR colonic OR caecal OR cecal) AND (microbiot* OR microbiom* OR microflora OR dysbios* OR metagenom* OR "16S rRNA")) OR "gut-bone axis" OR "gut bone axis") AND TITLE-ABS-KEY(osteoporos* OR osteopeni* OR "bone mineral density" OR BMD OR "bone mineral content" OR "bone mass" OR "bone loss" OR "bone resorption" OR "bone formation" OR "bone turnover" OR "bone remodelling" OR "bone remodeling" OR "bone microarchitecture" OR "bone strength" OR fracture* OR osteoclast* OR osteoblast* OR osteocyt*)

**Product and mediator supplement**

TITLE-ABS-KEY(osteoporos* OR osteopeni* OR "bone mineral density" OR BMD OR "bone mineral content" OR "bone mass" OR "bone loss" OR "bone resorption" OR "bone formation" OR "bone turnover" OR "bone remodelling" OR "bone remodeling" OR "bone microarchitecture" OR "bone strength" OR fracture* OR osteoclast* OR osteoblast* OR osteocyt*) AND TITLE-ABS-KEY(probiotic* OR prebiotic* OR synbiotic* OR postbiotic* OR "faecal microbiota transplant*" OR "fecal microbiota transplant*" OR FMT OR "Lactobacillus reuteri" OR "Limosilactobacillus reuteri" OR "Lactobacillus plantarum" OR "Lactiplantibacillus plantarum" OR "Lactobacillus paracasei" OR "Lacticaseibacillus paracasei" OR "Lactobacillus fermentum" OR "Limosilactobacillus fermentum" OR Bifidobacterium OR "Bacillus subtilis" OR "Akkermansia muciniphila" OR "Roseburia intestinalis" OR SBD111 OR CCFM1126 OR "short-chain fatty acid*" OR "short chain fatty acid*" OR SCFA* OR butyrate OR propionate OR acetate OR "bile acid*" OR "hyodeoxycholic acid" OR TGR5 OR FXR OR tryptophan OR indole* OR "aryl hydrocarbon receptor" OR AhR OR "bacterial extracellular vesicle*" OR "bacterial membrane vesicle*" OR "outer membrane vesicle*")

**Upstream context supplement**

TITLE-ABS-KEY(((gut OR intestin* OR gastrointestinal OR faecal OR fecal OR colonic OR caecal OR cecal) AND (microbiot* OR microbiom* OR microflora OR dysbios* OR metagenom* OR "16S rRNA")) OR "gut-bone axis" OR "gut bone axis") AND TITLE-ABS-KEY("calcium absorption" OR "mineral absorption" OR "intestinal permeability" OR "epithelial barrier" OR lipopolysaccharide* OR TLR4 OR Th17 OR Treg* OR "regulatory T cell*" OR "regulatory T-cell*" OR RANKL OR osteoprotegerin OR WNT10B OR serotonin OR "insulin-like growth factor" OR "IGF-1")

### S1.3.4 Cochrane Library

MeSH and title/abstract/keyword fields are combined. CENTRAL trial records and Cochrane Reviews have different evidence roles and should be reported separately. Clinical Answers are contextual summaries, not additional independent primary studies.

**#1 Microbiota MeSH**

[mh "Gastrointestinal Microbiome"]

**#2 Microbiota text**

(((gut OR intestin* OR gastrointestinal OR faecal OR fecal OR colonic OR caecal OR cecal) AND (microbiot* OR microbiom* OR microflora OR dysbios* OR metagenom* OR "16S rRNA")) OR "gut-bone axis" OR "gut bone axis"):ti,ab,kw

**#3 Skeleton MeSH**

[mh Osteoporosis] OR [mh "Bone Density"] OR [mh "Bone Remodeling"] OR [mh "Fractures, Bone"]

**#4 Skeleton text**

(osteoporos* OR osteopeni* OR "bone mineral density" OR BMD OR "bone mineral content" OR "bone mass" OR "bone loss" OR "bone resorption" OR "bone formation" OR "bone turnover" OR "bone remodelling" OR "bone remodeling" OR "bone microarchitecture" OR "bone strength" OR fracture* OR osteoclast* OR osteoblast* OR osteocyt*):ti,ab,kw

**#5 Products and mediators**

(probiotic* OR prebiotic* OR synbiotic* OR postbiotic* OR "faecal microbiota transplant*" OR "fecal microbiota transplant*" OR FMT OR "Lactobacillus reuteri" OR "Limosilactobacillus reuteri" OR "Lactobacillus plantarum" OR "Lactiplantibacillus plantarum" OR "Lactobacillus paracasei" OR "Lacticaseibacillus paracasei" OR "Lactobacillus fermentum" OR "Limosilactobacillus fermentum" OR Bifidobacterium OR "Bacillus subtilis" OR "Akkermansia muciniphila" OR "Roseburia intestinalis" OR SBD111 OR CCFM1126 OR "short-chain fatty acid*" OR "short chain fatty acid*" OR SCFA* OR butyrate OR propionate OR acetate OR "bile acid*" OR "hyodeoxycholic acid" OR TGR5 OR FXR OR tryptophan OR indole* OR "aryl hydrocarbon receptor" OR AhR OR "bacterial extracellular vesicle*" OR "bacterial membrane vesicle*" OR "outer membrane vesicle*"):ti,ab,kw

**#6 Upstream context**

("calcium absorption" OR "mineral absorption" OR "intestinal permeability" OR "epithelial barrier" OR lipopolysaccharide* OR TLR4 OR Th17 OR Treg* OR "regulatory T cell*" OR "regulatory T-cell*" OR RANKL OR osteoprotegerin OR WNT10B OR serotonin OR "insulin-like growth factor" OR "IGF-1"):ti,ab,kw

**#7 Core search**

(#1 OR #2) AND (#3 OR #4)

**#8 Product and mediator supplement**

(#3 OR #4) AND #5

**#9 Upstream context supplement**

(#1 OR #2) AND #6

## S1.4 Dates, filters and supplementary identification

Search date, publication date and database coverage are different fields. Year-only restrictions such as PY=1900-2026 or PUBYEAR < 2027 do not encode a day-specific publication cut-off. If a search was conducted on 20 July, that date remains the execution date; a later rerun may retrieve different records. Bibliographic issue dates can also differ from first online publication dates.

| **Database** | **Settings in the supplied record** | **Technical interpretation** |
| --- | --- | --- |
| PubMed | English[lang]; publication date through 2026/07/20 | The language filter concerns article language. It does not establish full-text access. Retain the publication-date restriction separately from the execution date. |
| Web of Science | DT=Article OR DT=Review; LA=English; PY=1900-2026 | The year range includes all of 2026. Article/review limits are additional filters, not part of the two-domain search logic. |
| Scopus | DOCTYPE(ar) OR DOCTYPE(re); LANGUAGE(english); PUBYEAR < 2027 | A year limit is not a 20 July cut-off. Broad labels such as Life Sciences are not a reproducible list of subject filters. |
| Cochrane Library | Publication Year 1996-2026; Reviews, Trials and Clinical Answers | A platform launch year should not automatically exclude older trial reports. Distinguish collections and the evidence role of each result. |

The search record describes backward reference checking and forward citation tracing from mechanistic papers, large cohorts, systematic reviews and recent trials. It also lists targeted searches of Frontiers in Immunology, Bone Research, Osteoporosis International, Bone, Journal of Bone and Mineral Research, Nature Communications, Cell Host & Microbe, Gut, Gut Microbes, JAMA Network Open and The Lancet. Journal searching supplements rather than replaces topic-based retrieval; journal prestige is not an eligibility criterion.

## S1.5 Eligibility source roles and evidence selection

| **Source role** | **Eligibility and interpretation** |
| --- | --- |
| Direct skeletal evidence | Primary human, animal or cellular studies linking a microbial exposure, intervention or mediator to a quantitative bone outcome. The study design and measured endpoint constrain the claim. |
| Upstream mechanistic support | Relevant mineral absorption, barrier, immune or endocrine findings without a skeletal endpoint. Such evidence supports a candidate link, not demonstrated osteoporosis efficacy. |
| Background and methods | Foundational bone biology, reporting standards and regulatory guidance used for definitions or interpretation. These sources need not satisfy primary skeletal-study criteria. |
| Evidence syntheses | Reviews and meta-analyses used for citation tracing, context and comparison of pooled estimates. Overlapping trials are not independent replications. |

For direct skeletal evidence, exclude unrelated bone diseases and studies without a relevant microbial exposure or quantitative bone outcome. These exclusions do not remove an explicitly identified upstream or background source. English-language full texts are required for detailed appraisal. Sample size informs precision and risk of bias rather than a fixed numerical exclusion threshold.

The selection record specifies independent title/abstract screening by two researchers, subsequent full-text assessment and discussion of disagreements, with third-person adjudication if needed. Search execution and review of the search strategy are separate roles from study selection.

Extraction for the synthesis records the study design, population or model, microbial exposure or assay, mediator and tissue compartment, skeletal endpoint, principal finding and interpretive limitation. Positive, null and discordant findings are considered on the same substantive criteria. S2 presents the selected evidence map; it is not an exhaustive systematic-review extraction table or a formal certainty grade.

Prospective registration and reporting of search counts are separate matters. The bibliography includes primary studies, syntheses, methods papers and other contextual sources, so its size is not a count of included primary studies.

## S1.6Technical documentation

PubMed search fields and history: https://pubmed.ncbi.nlm.nih.gov/help/

Web of Science advanced query builder: https://webofscience.zendesk.com/hc/en-us/articles/20130361503249-Advanced-Search-Query-Builder

Scopus advanced search syntax: https://www.elsevier.support/scopus/answer/how-can-i-best-use-the-advanced-search

Cochrane searching and selecting studies: https://www.cochrane.org/authors/handbooks-and-manuals/handbook/current/chapter-04

# S2 Selected evidence and clinical interpretation

The following map identifies what each selected study supports and the inference that still requires testing. It does not score or rank the complete literature.

| **Pathway / source** | **Model and directly supported link** | **Perturbation or measurement** | **Boundary / next test** |
| --- | --- | --- | --- |
| Sex-steroid deficiency ^[13]^ | Colonised versus germ-free mouse skeletal and inflammatory responses | Microbial status and specified probiotic intervention | Developmental effects of germ-free status; not all cortical or ageing loss is microbial |
| Immune trafficking ^[16]^ | Gut-associated T cells to marrow in an OVX model | Egress/recruitment interruption; TNF/Th17-related mechanisms | Model- and compartment-specific; no equivalent human tracking demonstrated |
| SCFA–osteoclast metabolism ^[20]^ | Direct precursor metabolic response and inhibited osteoclastogenesis | Receptor-deficient cells and glycolysis inhibition | Effect not assigned universally to GPR43; human exposure requires verification |
| Butyrate–Treg–WNT10B ^[21]^ | Treg-regulated CD8+ T-cell WNT10B and bone formation in mice | Treg depletion; adoptive-transfer loss of CD8-cell WNT10B | Not Treg-derived WNT10B; no clinical fracture efficacy |
| Butyrate and intermittent PTH ^[22]^ | Microbiota/butyrate-dependent component of mouse anabolic response | Microbial and metabolite perturbations | Interaction with human osteoporosis treatment remains to be tested |
| SCFA receptor counterexample ^[23]^ | Acetate-related osteogenic/adipogenic balance | *Gpr41/43* double knockout and marrow-cell experiments | Not a refutation of other ligands; receptor and cell context differ |
| HDCA–TGR5 ^[25]^ | Osteoclast maturation and mouse bone microstructure | HDCA exposure and TGR5 deletion; organism-only transfer insufficient | Does not establish FXR or a T-cell mediator; candidate human pharmacology |
| Indole–intestinal AhR ^[18]^ | Barrier and skeletal response in OVX mice; strongest localisation test for IAA | Intestinal epithelial AhR deletion; immune/IL-10 observations | No assumption of direct IL-10 transport; distinguish necessity from correlated immune changes |
| Bile-acid immune differentiation ^[24]^ | Mouse intestinal Th17/Treg responses | Defined bile-acid derivatives and molecular targets | No skeletal endpoint: upstream plausibility only |
| Control-donor FMT ^[17]^ | OVX mouse bone measures, junctional proteins and metabolites | Whole-community transfer | No Th17/Treg assay; no isolated mediator; junctional markers are not direct flux measures |
| Human immune association ^[15]^ | Peripheral immune frequencies, cytokines/LPS and stool profiles | Cross-sectional comparison of 77 women | No marrow sampling or cell trafficking; LPS not a standalone barrier assay |
| Human-to-mouse FMT ^[69]^ | Transferred donor-group-associated phenotype | Five human donors per group; antibiotic-treated mice | Recipient replication cannot substitute for independent donors |
| Ageing null model ^[43]^ | Age-related bone loss not dependent on microbiome in tested mice | Germ-free/colonisation and donor-age comparisons | Boundary of generalisation, not disproof of sex-steroid-dependent effects |
| FINRISK ^[44]^ | Baseline microbiome associated with later fracture | Prospective follow-up | Temporality without demonstrated skeletal mediator |
| *Roseburia* BEVs ^[32]^ | Vesicle/metabolite-related osteogenic and skeletal effects | Cells and animal model; metabolite administration | Metabolite sufficiency is not necessity for all vesicle effects |
| Engineered Akkermansia BEVs ^[62]^ | Defined cargo-associated mouse skeletal effects | Engineered delivery preparation | Not evidence of native physiological cargo delivery or human efficacy |
| Three-strain trial contrast ^[47, 49]^ | Shared strain accessions and daily count; different primary skeletal sites | Randomised trials with different populations and imaging endpoints | Neither exact replication nor a demonstrated host interaction |
| CCFM1126 clinical component ^[51]^ | DXA score changes in 31 retained participants | 10¹⁰ CFU/day for three months; linked clinical and experimental components | Within-group serum changes do not prove a between-group immune effect or clinical mediation |

## S2.1 Trial identity and outcome checks

The Nilsson and Gregori trials used *Limosilactobacillus reuteri* ATCC PTA 6475 but differed in age range and follow-up ^[46, 48]^. Nilsson randomised 90 women aged 75–80 and reported 70 completions. Gregori tested daily totals of 10⁹ or 10¹⁰ CFU in 239 early postmenopausal women over 24 months, with shared vitamin D supplementation. These contrasts motivate a host-context hypothesis; they do not demonstrate an age-by-treatment interaction.

The Jansson primary analysis required paired baseline and 12-month scans ^[47]^. Its strain accessions and daily viable count match those reported by Resciniti ^[49]^, but imaging sites and study settings differ. Both results are retained in Table 2; they should not be combined as if they measured one identical effect.

Resciniti reports 10¹⁰ CFU per capsule, one capsule daily for 12 months, in the intervention methods ^[49]^. Viability was checked during storage. The primary adjusted tibial estimate was −3.7 mg HA/cm³ (95% CI −6.9 to −0.4), favouring placebo; this is a site-specific imaging result, not an observed fracture event. The secondary lumbar-spine comparison had a raw P value of 0.042 but a false-discovery-rate-adjusted value of 0.056. Reporting the former alone as an independently confirmed spine effect would disregard multiplicity.

SBD111 supplied *Levilactobacillus brevis* SBS04254, *Lactiplantibacillus plantarum* SBS04260 and *Leuconostoc mesenteroides* SBS02455 at 3 × 10¹⁰ CFU/day each, plus *Pichia kudriavzevii* SBS04263 at 5 × 10⁹ CFU/day ^[50]^. Oligofructose and dried blueberry powder were each 640 mg/day. The total was 9.5 × 10¹⁰ CFU/day; 4.75 × 10¹⁰ is the twice-daily amount, not the daily total. Vitamin D was provided in all groups. The full-cohort primary lumbar-spine result was null, and subgroup findings do not establish overall efficacy.

The CCFM1126 clinical methods specify 5 × 10⁹ CFU per 2-g pack, twice daily ^[51]^. This human regimen must not be inferred from the animal CFU/mL experiments. Of 44 enrolled participants, 31 remained after withdrawals; wording about the allocation sequence is ambiguous. Results describe T- and Z-score changes rather than a clearly site-labelled absolute BMD treatment estimate. The cited BMD method identifies a DXA instrument but does not specify the skeletal site. Reported serum changes in vitamin D, OPG and TRACP-5b did not translate into significant post-intervention between-group differences; calcium was an exception. These distinctions limit a claim of human immune mediation, independently of the associated animal experiments.

## S2.2 Source locations and interpretation rules

| **Source** | **Location checked** | **Retained information** | **Boundary** |
| --- | --- | --- | --- |
| Jansson ^[47]^ | PubMed abstract, Methods and Findings | Analysis population, dose, primary endpoint and interval | Not a full-text risk-of-bias assessment |
| Resciniti ^[49]^ | Intervention methods; Tables 2–4 | Capsule dose; tibial effect; secondary multiplicity | Greater imaging loss is not demonstrated fracture harm |
| CCFM1126 ^[51]^ | Clinical product/BMD methods; clinical results | Human dose, retained sample, scores and serum contrasts | Clinical and animal mechanisms not merged |
| FDA ^[45]^ | Official announcement, 19 December 2025 | Endpoint qualification described in Section 4.6 | No class-wide validation of microbiota products |

“Established” refers to a general biological principle unless a treatment claim is explicitly identified. Preclinical support is tied to a named model and perturbed link. Human support retains the observational or randomised design label. “Unresolved” indicates that the assessed evidence does not establish the proposed link; it does not assert that no relevant study exists. These categories are not numerical grades. Claim checking focused on changed interpretations and selected primary estimates, with no assertion of independent raw-data verification .
